# Factors associated with directly observed treatment in tuberculosis/HIV coinfection cases in Porto Alegre, 2009-2013: A retrospective cohort

**DOI:** 10.1371/journal.pone.0222786

**Published:** 2019-10-02

**Authors:** Évelin Maria Brand, Maíra Rossetto, Karen da Silva Calvo, Gerson Barreto Winkler, Daila Alena Raenck da Silva, Bruna Hentges, Frederico Viana Machado, Erica Rosalba Mallmann Duarte, Lucas Cardoso da Silva, Samantha Correa Vasques, Luciana Barcellos Teixeira

**Affiliations:** 1 Department of Public Health, Universidade Federal do Rio Grande do Sul, Porto Alegre, Rio Grande do Sul, Brazil; 2 Department of Medicine, Universidade Federal da Fronteira Sul, Chapecó, Santa Catarina, Brazil; 3 Department of Professional Assistance and Guidance, Universidade Federal do Rio Grande do Sul, Porto Alegre, Rio Grande do Sul, Brazil; 4 Department of Social Medicine, Universidade Federal do Rio Grande do Sul, Porto Alegre, Rio Grande do Sul, Brazil; Chinese Academy of Medical Sciences and Peking Union Medical College, CHINA

## Abstract

**Background:**

TB/HIV coinfection is a serious public health issue in Brazil, and patients with coinfection have difficulty adhering to treatments. Directly observed treatment (DOT) has been recommended by the World Health Organization, considering the vulnerability of those affected. The purpose is to investigate the occurrence of DOT and associated factors compared to conventional treatment in Porto Alegre, Brazil.

**Methods:**

A retrospective cohort study was carried out with all patients with coinfection from 2009 to 2013 in the city of Porto Alegre, Brazil, the state capital with the highest rate of coinfection in Brazil. The data came from national health information systems. The dependent variable was the performance of DOT. Bivariate and multivariable models were used to determine factors associated with DOT. The percentage of cure and death was verified in a period of two years, comparing patients who received and did not receive DOT.

**Results:**

2,400 cases of coinfection were reported, with 1,574 males and 826 females and a mean age of 38 years ± 9.91 years. The occurrence of DOT was 16.9%. In the multivariable analysis, factors independently associated to DOT were the year (with greater chances of being received in 2012 and 2013), place of origin, non-white race (OR = 1.29, 95% CI = 1.08–1.54), cases of relapse (OR = 1.33; 95% CI = 1.03–1.73), readmission after abandonment (OR = 1.48, 95% CI = 1.20–1.83), transfer (OR = 2.04; 95% CI = 1.40–2.98), acid-fast bacilli (AFB) test with positive result in first sample (OR = 1.73, 95% CI = 1.24–2.42), alcohol abuse (OR = 1.39; 95% CI = 1.16–1.67), and mental disorders (OR = 1.83; 95% CI = 1.38–2.44.) Of the 532 cases of death, occurring in two years, 10.2% were in patients who underwent DOT and 89.8% in patients who did not undergo DOT (p<0.001). O percentual de óbitos em pessoas que receberam DOT foi de 13% e o percentual de óbitos para pessoas que receberam tratamento convencional foi de 24%.

**Conclusions:**

There was an increase in the percentage of DOT over the years in the scenario studied, and the predictors for DOT were related to social vulnerability. In relation to death within two years, a lower proportion was found in patients who underwent DOT, suggesting a protective effect of the strategy.

## Introduction

Tuberculosis (TB) and AIDS are considered to be neglected diseases due to their association with poverty, the vulnerability of individuals and the contribution to health inequalities [1,2]. WHO estimates that worldwide in 2017 there were 920,000 new cases of TB among those infected with HIV, equivalent to 9.2% of the 10 million new cases of tuberculosis diagnosed in the same year. Among people living with HIV/AIDS, 0.3 million died of tuberculosis in 2017 [3].

Brazil is the country with the highest number of new cases of tuberculosis in the Americas, with a total of 91,000 estimated cases in 2017, equivalent to 32% of all estimated cases of the disease in the Americas [4,5]. As of 2016, 6,501 people with HIV developed tuberculosis [6]. In Porto Alegre, coinfection cases accounted for 24.1% of new cases of tuberculosis in 2016 [6]. Identification and treatment of TB/HIV coinfection are essential to reduce the number of cases of morbidity and mortality for both diseases. As for TB treatment in Porto Alegre, only 61.4% of the new cases of the disease in 2015 were cured, and the percentage of treatment abandonment was 16.3% of the new cases [5].

Non-adherence to drug therapy is the major barrier to treatment success as abandonment is a factor for the follow-up of disease transmission, with an impact on relapse rates of coinfection and increasing drug resistance [7–9]. Risk factors for abandonment include being male between the ages of 30 and 39, being illiterate or not having completed high school, having family conflicts, not living in the central districts of cities, being a user of licit and/or illicit drugs, and having previously been treated for TB [10].

Against this background, the challenge of public policies is to guarantee the adherence of people with coinfection to the treatments. The main strategy of the World Health Organization to promote adherence is the Directly Observed Therapy Short Course (DOTS), a strategy proposed since 1991 and made up of five components: political commitment, case detection through sputum smear microscopy; Directly Observed Treatment (DOT); a regular supply of medicines; and a system of notification and follow-up of cases. In 2015, in order to control TB, the WHO set targets through the Stop TB Partnership, recommending the implementation of DOTS [2]. In Brazil, the need for DOT is assessed by the primary or secondary care health team and performed by the primary care health team. Conventional treatment for co-infection cases consists of monthly consultations with primary and secondary care teams, without direct observation of medication intake. The DOT is directed to the treatment of tuberculosis and performed by the nursing staff. It consists of the daily observation of medication intake for at least the first 30 days of treatment and may continue until the end of treatment. [11].

In scenarios with a high proportion of abandonment, treatment failure and frequency of adverse events in patients with coinfection, it is necessary to intensify strategies to promote effective treatment [11]. Regarding coinfection, international recommendations emphasize the reduction of multidrug resistance (MDR) and mortality, with timely diagnosis and DOT as the main focus [2]. In a Brazilian study that analyzed cases of TB-HIV co-infection reported in the Brazilian information system (SINAN) between 2001 and 2011, it was seen that DOT gave protection against TB treatment abandonment and death [12]. In Rio de Janeiro, a 2018 study identified an association between TB treatment outcome and clinical and sociodemographic factors. However, this study did not analyze cases of TB / HIV co-infection [13]. In Porto Alegre, a recent study showed that the occurrence of hospitalizations and deaths is high among people with both HIV and TB [14]. Thus, the aim was to investigate the occurrence of DOT and associated factors compared to conventional treatment.

## Materials and methods

### Study design

This is a retrospective cohort study, classified as a dynamic or open cohort [15]. Participants were included in the study at any time between 2009 and 2013. In this type of cohort, there is no fixed recruitment period for individuals, and in view of the possibility of recurrence due to tuberculosis, patients entered the cohort if they were diagnosed and registered in the national surveillance system. Thus, the study was based on cases of coinfection, and the case definition corresponds to the entry in the Epidemiological Surveillance System. Considering that the follow-up period was up to two years, closure occurred in 2015 in order to ensure the equal follow-ups of all patients.

### Study population

The participants of this study are all patients with a diagnosis of TB/HIV coinfection in the city of Porto Alegre, aged over 18 years, and registered in the national health surveillance system from 2009 to 2013. The sample is expected to correspond to the totality of patients with coinfection, since TB and HIV are compulsory notification diseases in Brazil and, therefore, all patients diagnosed should be notified by the national surveillance system. Nevertheless, the existence of underreporting in this system should be considered, and so SINAN TB cases can be considered a proxy of TB cases in Brazil.

### Study procedures

The dependent variable of the study was the achievement of directly observed treatment. The sociodemographic variables were: sex (female and male), years of education completed, age and race (white and not white). From the clinical point of view, we analyzed: entry into the surveillance system, bacilloscopy examination (positive, negative or not performed), the occurrence of other negative factors (alcohol abuse and mental disorders), and status at completion that were obtained from the national tuberculosis notification data bank (SINAN-TB). The type of entry was classified into new case (never treated), relapse (patient cured and infected once again by tuberculosis), return after default (patient who interrupted the treatment and is about to resume it) or transfer (patients who had been transferred from another town or city to the city of Porto Alegre). The new case category was defined as the reference category for analysis. The variable status at completion refers to what happened after the tuberculosis treatment was monitored by the national surveillance system, that is: cure, default, transference, death or multiresistance. In this same bank, the occurrence of coinfection was identified.

The DOT Indication variable considered the indication of treatment at the moment of the notification/diagnosis of the case. The indication of DOT is a clinical judgment made by the professional who completed the notification form.

For the reliability of the study data, information on the total of coinfected and the number of deaths were also checked in other national databases. Following the organization of the study database from the SINAN-TB system [16], all cases of TB whose HIV test was negative or in progress were checked in the national HIV/AIDS notification system (SINAN-HIV/AIDS) [16].

Patient follow-up occurred until the closure of the case in the tuberculosis surveillance system, which occurred after 12 months of follow-up, with the patient’s classification as cure, abandonment, death, multidrug-resistant TB, or transfer. For more reliable information on TB/HIV mortality, all cases of coinfection were subsequently checked in the National Mortality Information System (SIM) [17], and 3 cases were found not to have been included in the tuberculosis surveillance system as death within 12 months of follow-up, but whose death had actually occurred within 12 months due to TB/HIV.

Despite being a study with secondary databases, patient identification was required for verification in different databases. The patient’s full name, mother’s name and registration number in the public health system were used as an identification procedure, and it was necessary to confirm these three data sets to consider that it was the same case in the tuberculosis, HIV/AIDS, and SIM databases. There were only 2 SINAN cases where the patients were not reported as having died of tuberculosis and whose full name and mother’s name were identical in the SIM, but given that the health card number was different, they were not included as deaths.

In terms of the place of residence, the patients were classified by the District Health Authorities (DHA). The DHA are configured as administrative structures for the management of the local health network and may include more than one health district. There are eight DHA in Porto Alegre: Centro (CEN), North/Baltazar Axis (NEB), East/Northeast (LENO), Gloria/Cruzeiro/Cristal (GCC), South/Center-South (SCS), Partenon/Lomba do Pinheiro (PLP), Restinga/South End (RES), and Northwest/Humaitá/Navegantes/Ilhas (NHNI), some of which present greater social vulnerability, depending on the population profile [18].

After the classification of the patients by DHA, according to their address, the identification information of the cases (full name, mother’s name, health card number, and address) were erased, creating a numerical code for each case in the study, aiming to maintain the anonymity of individuals, according to the determination of the research legislation in force in Brazil [19].

### Statistical analyses

SPSS 21.0 (IBM) was used to perform the statistical analyses. The T-Student test was carried out to compare the continuous age variable with DOT. Descriptive statistics were used to present the characteristics of the participants and the occurrence of DOT. Comparisons were made between patients who received and did not receive DOT regarding characteristics when entering the surveillance system and at the closure, considering the possibility of cure and death, through a homogeneity of proportions test based on Pearson’s chi-square statistic. The factors associated with DOT were analyzed by bivariate and multivariable models that estimated the odds ratios by means of logistic regression with a 95% confidence interval. All variables were considered statistically significant (p-value of <0.05).

### Ethics statement

This study complied with the guidelines of the Resolution 466/2012 of the Brazilian National Health Council. The research project was approved by the Ethics and Research Committee of the Federal University of Rio Grande do Sul (UFRGS) and by the Ethics Committee of the Municipal Government of Porto Alegre, opinions 952907 and 939250, respectively. Since this is a linkage study of national databases, patients needed to be identified for the linkage procedure to succeed. This was informed in the research protocol to the ethics committee, which approved the study, waiving the need for a term of consent.

## Results

From 2009 to 2013, 8,813 TB cases were found in the SINAN-TB database. Of these, 75 were duplicate cases, with double notification, which were excluded. Of the total, 2,419 cases had a diagnosis of HIV at the time of TB notification, and therefore were considered coinfected.

Ten cases of HIV/AIDS patients that were not initially coinfected in SINAN-TB were found in the national HIV/AIDS notification system. These patients were included in the study database with the other people with both HIV and TB and were excluded from the statistical analysis, as the fact that they did not present coinfection at the time of definition of DOT could mean that they had not been listed for DOT. Thus, 2,419 cases of coinfection were found. However, for 19 cases there was no information on the performance of DOT in SINAN-TB, and these were excluded. The final sample was 2,400 cases of TB/HIV coinfection.

Of 2400 cases, DOT was performed in 17% (406). In relation to the year of registration, of the 398 cases registered in 2009, 9.3% performed DOT. In 2013, of the 575 registers, 31.7% performed DOT (<0.001). Considering the origin, the percentage of cases with DOT was from 9.5% to 26% (<0.001). Among whites the performance of DOT was 14.6%, and among non-whites 20% (<0.001). The percentage of DOT varied according to schooling, being more frequent in people with ≤7 years (p = 0.021) (Table 1).

**Table 1:** Sociodemographic characteristics and numbers receiving directly observed therapy among cases of TB/HIV coinfection in Porto Alegre, 2009–2013.

Characteristics	Total^b^	Receiving DOT^a^		p-value
		Yes	No	
**Reporting year**				<0.001*
2009	398 (16.6%)	37 (9.3%)	361 (90.7%)	
2010	408 (17%)	44 (10.8%)	364 (89.2%)	
2011	494 (20.6%)	48 (9.7%)	446 (90.3%)	
2012	525 (21.9%)	95 (18.1%)	430 (81.9%)	
2013	575 (24%)	182 (31.7%)	393 (68.3%)	
**DHA**^c^				< 0.001*
CEN	427 (17.8%)	111(26%)	316 (74%)	
NHNI	158 (6.6%)	17 (10.8%)	141 (89.2%)	
NEB	263 (11%)	26 (9.9%)	237 (90.1%)	
LENO	376 (15.7%)	57 (15.2%)	319 (84.8%)	
GCC	285 (11.9%)	40 (14%)	245 (86%)	
SCS	168 (7%)	16 (9.5%)	152 (90.5%)	
PLP	550 (22.9%)	112 (20.4%)	438 (79.6%)	
RES	171 (7.1%)	27 (15.8%)	144 (84.2%)	
**Age**	38±9.9	37.8±9.2	38±10	0.766***
**Race/ethnicity**				<0.001**
White	1346 (56.3%)	196 (14.6%)	1.150 (85.4%)	
Non-white^d^	1046 (43.7%)	209 (20%)	837 (80%)	
**Sex**				0.457**
Male	1574 (65.6%)	273 (17.3%)	1.301 (82.7%)	
Female	826 (34.4%)	133 (16.1%)	693 (83.9%)	
**Years of education completed**				0.021*
≤7 years	1536 (69.2%)	282 (18.4%)	1254 (81.6%)	
8–11 years	625 (28.2%)	88 (14.1%)	537 (85.9%)	
≥12 years	59 (2.7%)	6 (10.2%)	53 (89.8%)	
**Total**	**2400 (100%)**	**406 (17%)**	**1994 (83%)**	

*Proportion homogeneity test based on Pearson’s chi-square statistic or **Fischer’s exact test.

***T-student test for independent samples.

^a^DOT = Directly observed therapy.

^b^Total data may differ due to the possibility of the non-response by participants.

^c^District Health Authorities (DHA) = CEN: Centro, NEB: North/Baltazar Axis, LENO: East/Northeast, GCC: Gloria/Cruzeiro/Cristal, SCS: South/Center-South, PLP: Partenon/Lomba do Pinheiro, RES: Restinga/South End, NHNI: Northwest/Humaitá/Navegantes/Ilhas.

^d^Non-white population is the sum of the colored population (black and brown), alongside Asians and indigenous people.

Regarding the type of entry, a lower percentage of DOT was observed in new cases (12.7%), and a higher percentage among cases of transference (35.7%) (p <0.001). DOT was received in 24.3% of the cases with alcohol abuse and in 14.4% of cases without alcohol abuse (p <0.001). In 35.1% of the cases with mental health problems, DOT was received (p <0.001). DOT was received in 17.4% of the cases of cure, 18.6% of the cases of default, 10.2% of the cases that died, 20.7% of the cases that were transferred, and in 35.5% of the Multidrug-resistant TB cases (p <0.001). The percentage of deaths in people who received DOT was 13% (54/406) and the percentage of deaths in people who received conventional treatment was 24% (478∕1994) (Table 2).

**Table 2:** Clinical and follow-up characteristics and the occurrence of directly observed therapy among cases of TB/HIV coinfection in Porto Alegre, 2009–2013.

Characteristics	Total^b^	Receiving DOT^a^		p-value
		Yes	No	
**Type of entry**				<0.001*
New case	1382 (57.6%)	175 (12.7%)	1207 (87.3%)	
Relapse	347 (14.5%)	65 (18.7%)	282 (81.3%)	
Readmission after default	615 (25.6%)	146 (23.7%)	469 (76.3%)	
Transfer	56 (2.3%)	20 (35.7%)	36 (64.3%)	
**Chest X-ray**				0.279*
Suspicion	2137 (89.2%)	366 (17.1%)	1771 (82.9%)	
Normal	56 (2.3%)	9 (16.1%)	47 (83.9%)	
Other pathology	29 (1.2%)	1 (3.4%)	28 (96.6%)	
Not performed	173 (7.2%)	29 (16.8%)	144 (83.2%)	
**1**^**st**^ **sputum AFB test**^c^				0.002*
Positive	1414 (58.9%)	268 (19%)	1146 (81%)	
Negative	687 (28.6%)	105 (15.3%)	582 (84.7%)	
Not performed	299 (12.5%)	33 (11%)	266 (89%)	
**2**^**nd**^ **sputum AFB test**^c^				0.003*
Positive	1001 (41.7%)	195 (19.5%)	806 (80.5%)	
Negative	661 (27.6%)	112 (16.9%)	549 (83.1%)	
Not performed	736 (30.7%)	98 (13.3%)	638 (86.7%)	
**Indication for DOT**^a^				<0.001**
Yes	618 (25.8%)	318 (51.5%)	300 (48.5%)	
No	1778 (74.2%)	87 (4.9%)	1691 (95.1%)	
**Alcohol abuse**				<0.001**
Yes	613 (25.6%)	149 (24.3%)	464 (75.7%)	
No	1784 (74.4%)	257 (14.4%)	1527 (85.6%)	
**Diabetes**				0.508**
Yes	66 (2.8%)	13 (19.7%)	53 (80.3%)	
No	2330 (97.2%)	392 (16.8%)	1938 (83.2%)	
**Mental disorders**				<0.001**
Yes	97 (4.1%)	34 (35.1%)	63 (64.9%)	
No	2297 (95.9%)	370 (16.1%)	1927 (83.9%)	
**Status at completion**				<0.001*
Cure	852 (35.7%)	148 (17.4%)	704 (82.6%)	
Default	855 (35.8%)	159 (18.6%)	696 (81.4%)	
Death	532 (22.2%)	54 (10.2%)	478 (89.8%)	
Transfer	87 (3.6%)	18 (20.7%)	69 (79.3%)	
MDR-TB^d^	62 (2.6%)	22 (35.5%)	40 (64.5%)	
**Total**	**2400 (100%)**	**406 (17%)**	**1994 (83%)**	

Homogeneity of proportions test based on Pearson’s chi-square statistic* or Fischer’s exact test**.

^a^DOT = Directly observed therapy,

^b^total data may differ due to the possibility of the non-response by participants,

^c^AFB test = acid-fast bacilli test,

^d^MDR-TB = Multidrug-resistant TB.

The 1^st^ sputum AFB test in DOT group was positive in 19% of cases, negative in 15.3%, and not performed in 11%. In cases in which there was no DOT, this test was positive in 81%, negative in 84.7%, and not performed in 89% (p = 0.002). The 2^nd^ sputum AFB test was positive in 19.5%, negative in 16.9% and not performed in 13.3% of the cases that underwent DOT. In the group without DOT, this test was positive in 80.5% of the cases, negative in 83.1%, and not performed in 86.7% (p = 0.003).

Alcohol abuse was associated in 24.3% of cases with DOT and in 75.7% of cases compared to conventional treatment (p<0.001). Occurrence of mental disorders was associated in 35.1% of the group that had DOT and in 64.9% of the group without DOT (p<0.001).

The indication for DOT occurred in 51.5% of the group with DOT and 48.5% in the group without DOT (p<0.001). In the group for which DOT was received, 17.4% of the cases evolved to cure, 18.6% to default, 10.2% to death, 20.7% to transfer, and 35.5% to MDR-TB. In the group for which DOT was not received, 82.6% evolved to cure, 81.4% to default, 89.8% to death, 79.3% to transfer, and 64.5% to MDR-TB (p<0.001).

Chest X-ray (p = 0.279), diabetes (p = 0.508) and hospitalization (p = 0.231) did not present a statistically significant difference within the groups studied.

Table 3 presents the factors associated with directly observed treatment. A positive association was observed in the crude and adjusted models between year and DOT, being significant for the years 2012 (p = 0.007) and 2013 (p<0.001). Considering the place of origin in the city, there was an association between the PLP and CEN locations and performance of DOT in the crude and adjusted models. Patients at the CEN location had 2.29 (95% CI = 1.38–3.76) times more chance of performing DOT, while PLP patients had 1.72 (95% CI = 1.04–2.85) times more chance of performing DOT, when compared to SCS, the area with the lowest social vulnerability in the city. Non-white patients presented 1.29 (95% CI = 1.08–1.54) times more chance of DOT when compared to whites. Years of education completed was not associated with the outcome in the final model.

**Table 3:** Factors associated with directly observed therapy in patients with TB/HIV coinfection in Porto Alegre, 2009–2013.

Characteristics	Receiving DOT^a^		p-value
	Crude OR^b^ (95% CI)	Adjusted OR^c^ (95% CI)	
**Reporting year**			
2009	1.00	1.00	
2010	1.16 (0.77–1.76)	1.16 (0.77–1.74)	0.489
2011	1.05 (0.69–1.57)	0.94 (0.62–1.42)	0.759
2012	1.95 (1.36–2.78)	1.64 (1.14–2.35)	0.007
2013	3.41 (2.45–4.73)	2.89 (2.07–4.05)	<0.001
**DHA**^d^			
CEN	2.73 (1.67–4.47)	2.28 (1.38–3.76)	0.001
NHNI	1.13 (0.59–2.16)	1.13 (0.60–2.13)	0.703
NEB	1.04 (0.57–1.88)	0.87 (0.48–1.59)	0.659
LENO	1.59 (0.94–1.69)	1.29 (0.76–2.19)	0.343
GCC	1.47 (0.85–2.55)	1.34 (0.78–2.31)	0.296
SCS	1.00	1.00	
PLP	2.14 (1.30–3.51)	1.72 (1.04–2.85)	0.033
RES	1.66 (0.93–2.96)	1.72 (0.97–3.06)	0.064
**Race/ethnicity**			
White	1.00	1.00	
Non-white	1.37 (1.15–1.64)	1.29 (1.08–1.54)	0.006
**Years of education completed**			
≤7 years	1.81 (0.84–3.88)	1.52 (0.70–3.27)	0.289
8–11 years	1.39 (0.63–3.03)	1.29 (0.59–2.79)	0.524
≥12 years	1.00	1.00	
**Type of entry**			
New case	1.00	1.00	
Relapse	1.48 (1.14–1.92)	1.33 (1.03–1.72)	0.026
Readmission after default	1.88 (1.54–2.29)	1.48 (1.20–1.83)	<0.001
Transfer	2.82 (1.93–4.12)	2.04 (1.40–2.98)	<0.001
**1**^**st**^ **sputum AFB test**			
Positive	1.72 (1.22–2.41)	1.73 (1.24–2.42)	0.001
Negative	1.00	1.00	
Not performed	1.39 (0.96–1.99)	1.48 (1.04–2.85)	0.032
**Alcohol abuse**			
Yes	1.69 (1.41–2.02)	1.39 (1.16–1.67)	<0.001
No	1.00	1.00	
**Mental disorders**			
Yes	2.18 (1.63–2.89)	1.83 (1.38–2.44)	<0.001
No	1.00	1.00	

^a^DOT = Directly observed therapy,

^b^univariate models,

^c^multi-variable model by the logistic regression method,

^d^District Health Authorities (DHA) = CEN: Centro, NEB: North/Baltazar Axis, LENO: East/Northeast, GCC: Gloria/Cruzeiro/Cristal, SCS: South/Center-South, PLP: Partenon/Lomba do Pinheiro, RES: Restinga/South End, NHNI: Northwest/Humaitá/Navegantes/Ilhas.

Considering the classification of type of entry in the surveillance system, the patients were compared with the new cases (reference category). Patients classified as relapsing presented 1.33 (95% CI = 1.03–1.73) times more chance of performing DOT, patients classified as readmissions after default had 1.48 (95% CI = 1.20–1.83) times more chance of DOT, and patients with transfer presented 2.04 (95% CI = 1.40–2.98) times more chance of DOT. In relation to the AFB test, patients with a positive result in the first sample presented 1.73 (95% CI = 1.24–2.42) times more chance of performing DOT, and those without the examination had 1.48 (95% CI = 1.04–2.85) times more chance of DOT. Patients with alcohol abuse presented 39% (OR = 1.39; 95% CI = 1.16–1.67) more DOT when compared to patients without alcohol abuse. Patients with mental disorders presented 83% more DOT (OR = 1.83; 95% CI = 1.38–2.44) when compared to patients without mental disorders.

## Discussion

DOT is a strategy advocated by the WHO for coping with tuberculosis, particularly in cases of drug resistance and coinfection with HIV [20]. Comparing health outcomes in patients who perform and do not perform DOT makes it possible to highlight the impact of this strategy. In 2015, a systematic review with 11 clinical trials including 5,662 participants showed that there were virtually no differences in cure rates when comparing patients with DOT to other methods. In 2018, a new systematic review identified 7,729 articles, of which 129 evidenced that DOT improves adherence compared to other treatment methods [21]. TB/HIV coinfection is a double burden of diseases, with high incidence and mortality rates in Brazil, cases of TB/HIV coinfection have been prioritized for DOT, due to greater vulnerability to treatment abandonment, MDR-TB, and mortality [11,22,23].

Between 2007 and 2011, a study carried out in the same scenario of this research found that among the new cases of TB, 10.7% received DOT, and among all TB cases, the percentage of DOT was 14.7% [22]. In Porto Alegre, in 2016, 15.3% of the cases of pulmonary TB performed DOT. In the state of Rio Grande do Sul (RS), whose capital is Porto Alegre, this figure reached 17.2%, and in Brazil, 36.3% [4]. In countries where the burden of tuberculosis is immense, despite the increase in the number of cases, there is evidence of an increase in DOT, with a positive impact on the scenario of illness [24,25].

Between 2009 and 2013, there was an increase in the number of notifications of TB/HIV coinfection and in the performance of DOT. While the proportion of coinfection increased by approximately 8% over 5 years, the proportion of subjects performing DOT increased from 9.3% to 31.7% in the same period. In this sense, greater odds of performing DOT were observed in the last two years of the cohort, i.e., 2012 (p = 0.007) and 2013 (p<0.001). This finding may be related to the decentralization policy of tuberculosis care, instituted in 2011. In this policy, primary care began performing contact evaluation and referring patients with diagnostic difficulties and people living with HIV to the Reference Centers in Tuberculosis (CRTB), as well as receiving users referred from the CRTB or other specialized services for the performance of shared DOT [18,26]. Thus, even if coinfection cases refer to CRTB care, this care is not exclusive to the referral service but is recommended to be shared with Primary Care. This coping strategy aims to reduce the high rates of TB that exist in Brazil and improve adherence to antiretroviral therapy. Recently, a study in Ethiopia also recommended decentralizing treatment as a strategy to increase adherence [25].

Other factors may have contributed to the expansion of DOT. Although in Porto Alegre, it tends to be lower than in the state of Rio Grande do Sul (42.08%), and Brazil (56.37%), between 2009 and 2013, the Family Health Strategy (FHS) coverage increased from 22.4% to 31.2% [27]. Through the implementation of FHS, there are more health professionals throughout the city of Porto Alegre.

Moreover, because of the high incidence of TB/HIV coinfection in Porto Alegre, the state capital in Brazil with the highest incidence of TB and HIV, the primary care system has deemed it opportune to discuss the decentralization of HIV/AIDS care and carry out the proposal for decentralization of tuberculosis testing and treatment [28].

The logistic regression model showed a 2.28 fold higher chance of performing DOT for patients at the CEN site. The high vulnerability index of the district and the presence of the population in situations of homelessness are highlighted. The region also presents high rates of violence, mortality due to external causes, high numbers of families living in extreme poverty, and population over crowding in large favelas [18]. All these factors increase the vulnerability of the individuals in this situation [29,30]. Notwithstanding, the district has a health center that is historically considered one of the pioneers in tuberculosis treatment [18]. Sex and age were not associated with the outcome, as in other studies that point out that these are not decisive factors for the performance of DOT [31–33]. In terms of sociodemographic variables, data on race and schooling show the vulnerability associated with DOT. Although the highest proportion of cases of coinfection involved white individuals, there was a greater indication of DOT for non-whites. The adjusted regression model showed that, despite the differences in proportion, non-white patients presented a 29% greater chance of DOT when compared to whites. A recent study conducted in the same scenario showed that the need for hospitalization for TB treatment is more frequent in non-white patients living in poverty [34]. In our study, schooling was used as a proxy for the economic situation, and, although schooling was not associated with DOT, the lower the schooling, the greater the percentage of DOT, a trend already observed in other studies [33,35].

Reinforcing the vulnerability pointed out by other authors that associate the occurrence of alcohol abuse and mental illness with the difficulty of adherence to treatments [36,37], our study, observed that patients with alcohol abuse presented a 39% higher level of DOT performance than those without a history of abusive use. Moreover, those with mental disorders presented a 83% greater performance of DOT.

Considering the admission classification in SINAN, patients with readmission after abandonment presented 1.48 times more chance of performing DOT when compared to new cases. Relapse cases presented 1.33 times more chance of DOT, and transfer cases presented 2.04 times more chance of DOT. Treatment abandonment is one of the main challenges in addressing TB/HIV coinfection. The main consequences are the development of MDR-TB, increased hospitalizations, occurrence of mortality, and higher treatment costs [20,38,39].

Regarding the AFB sputum test for the diagnosis of TB, the result of the positive bacilloscopy was more frequent in DOT group in the 1st sputum collection. The maintenance of the tuberculosis transmission chain is related to the bacilliferous cases of the disease [40], evidencing the need for intervention in these cases. It is possible that the finding of 1.73 times greater chance of performing DOT in patients with positive AFB in the 1^st^ test is related to the great concern among health professionals of the transmissibility of TB [40]. It is also worth noting that sputum smear microscopy is the most widely used method for the diagnosis of the disease, and, when correctly performed, allows the detection of 60–80% of cases [40].

The difference of proportions found in the indication of DOT was already expected. Nevertheless, it is noted that 48.5% of the cases indicated did not perform DOT, while 4.9% of the cases that had no indication did perform DOT. These two groups require further investigation regarding their follow-up and outcomes as these findings are possibly related to the difficulties of the health services in offering DOT to those who are indicated at the start of follow-up [41,42].

Regarding the closure situation, although the highest percentage of cure was in the group without DOT, the highest proportion of MDR-TB was found in the group without DOT (64.5% versus 35.5%), a higher proportion of abandonment (81.4%) in the group without DOT [43–45], and among those who died 89.8% received conventional treatment and e 10.2% received DOT, demonstrating the effectiveness of this follow-up, as already indicated in other studies [46,47].

The main limitation of this study is the design adopted. In the retrospective cohort studies, the data were already recorded before the study. Despite this, it should be noted that in this study, the data used came from the national health information systems, in which compulsory notification phenomena are registered (tuberculosis & AIDS and death) and, therefore, have high reliability.

## Conclusion

The study evidenced an increase in DOT over the five years of the cohort. Higher odds of performing DOT were observed in the CEN district, in the cases with low schooling, among non-whites, in situations of relapse and readmission after abandonment, in cases with a positive first sputum sample, and in those that presented alcohol abuse and mental disorders as comorbidities, thereby indicating the context of vulnerability of coinfection. The association of these variables with the performance of DOT may indicate a greater ability of health professionals to identify subjects with a potential risk of treatment abandonment.

The study showed that, among patients who died, most had not received DOT, thus suggesting a protective effect. Therefore, as the reduction in mortality related to tuberculosis and HIV/AIDS is one of the major concerns when addressing coinfection, DOT has emerged as an effective strategy and should be expanded within health teams.

## Supporting information

S1 Dataset DOTS co-infection. (XLSX)
